# Sex-related differences in the association between waist circumference and bone mineral density in a Korean population

**DOI:** 10.1186/1471-2474-15-326

**Published:** 2014-10-02

**Authors:** Lian-Hua Cui, Min-Ho Shin, Sun-Seog Kweon, Jin-Su Choi, Jung-Ae Rhee, Young-Hoon Lee, Hae-Sung Nam, Seul-Ki Jeong, Kyeong-Soo Park, So-Yeon Ryu, Seong-Woo Choi

**Affiliations:** Department of Preventive Medicine, Qingdao University Medical College, Qingdao, China; Department of Preventive Medicine, Chonnam National University Medical School, 5 hak-dong, Dong-gu, 501-746 Gwangju City, South Korea; Jeonnam Regional Cancer Center, Chonnam National University Medical School, Hwasun Hospital, Hwasun, Republic of Korea; Department of Preventive Medicine & Institute of Wonkwang Medical Science, Wonkwang University College of Medicine, Iksan, Republic of Korea; Department of Preventive Medicine, Chungnam National University Medical School, Daejeon, Republic of Korea; Department of Neurology & Research Institute of Clinical Medicine, Chonbuk National University-Biomedical Research Institute of Chonbuk National University Hospital, Jeonju, Republic of Korea; Department of Preventive Medicine, Seonam University College of Medicine, Namwon, Republic of Korea; Department of Preventive Medicine, Chosun University Medical School, Gwangju, Republic of Korea

**Keywords:** Abdominal obesity, Waist circumference, Bone mineral density

## Abstract

**Background:**

Large waist circumference is linked to poor health. Investigations of the relationship between waist circumference, as an index of abdominal fat, and bone mineral density (BMD) have yielded inconsistent results. We investigated the association between abdominal obesity measured using waist circumference and BMD in a large-scale population-based study.

**Methods:**

We enrolled 8981 Korean (3592 males and 5389 females) community-dwelling individuals aged ≥50 years from 2007 to 2010. BMD was measured using dual-energy X-ray absorptiometry at lumbar spine and femoral neck skeletal sites. A multiple linear regression analysis was used to evaluate the relationship between waist circumference quartiles and BMD after adjusting for age, height, weight, and regular exercise.

**Results:**

The adjustment for age, height, weight, and regular exercise revealed a negative linear association between quartile of waist circumference and BMD at the femoral neck and lumbar spine sites in males and females. Waist circumference was more strongly correlated with BMD in males than in females. Although the correlations were slightly attenuated following further adjustment for percent body fat, they remained statistically significant.

**Conclusions:**

Our results revealed that waist circumference is independently and inversely associated with BMD after adjusting for age, weight, height, regular exercise and percent body fat, suggesting that waist circumference is a potential predictor of osteoporosis in middle-aged and older Korean males and females.

**Electronic supplementary material:**

The online version of this article (doi:10.1186/1471-2474-15-326) contains supplementary material, which is available to authorized users.

## Background

The increasing prevalence of osteoporosis and obesity has created a significant health problem worldwide. The proportion of elderly people in Korea was 7.4% in 2000 and is expected to reach 15.1% in 2020. The public health burden of osteoporotic fractures is expected to rise as the aging population increases. Furthermore, the overall prevalence of obesity (body mass index, (BMI) >25 kg/m^2^) in Korean adults increased from 25.8 to 32.8% between 1998 and 2012. Obesity has a considerable impact on health and increases the risk of several chronic diseases, including insulin resistance, metabolic syndrome, and type 2 diabetes mellitus. Abdominal obesity is associated with increased all-cause, cardiovascular[1–4], cancer mortality[4–6], and overall mortality. Abdominal fat accumulation may be a predictor of a pro-inflammatory state[7–9], and recent data have demonstrated overlapping pathways between bone biology and inflammatory processes[10–14]. Thus, investigation of the relationship between osteoporosis and abdominal obesity in the aging population is warranted.

Previous studies using BMI as an indicator of overall obesity found that higher BMI was correlated with high bone mass, reductions in body weight were associated with bone loss, and that the positive association between body weight or BMI and bone mineral density (BMD) was related to a weight-bearing effect on bone, leading to the conclusion that obesity was protective against fracture[15–17]. However, subsequent studies did not confirm the protective effect of obesity on osteoporosis, with reports of both significant negative[18–22] and positive[23–26] correlations between body weight and bone health. However, central obesity was suggested to be more relevant to bone health than general obesity reflected by BMI; thus, differences in the relationships between surrogate and direct measures of central adiposity and BMD were proposed to underlie the contradictory findings.

Waist circumference is frequently used as a simple, inexpensive measure of central obesity in population-based studies. However, the results of previous investigations of the association between waist circumference and BMD have been inconsistent[22, 23, 25, 27, 28]. Thus, we investigated the association between abdominal obesity—as measured by waist circumference—and BMD after adjusting for age, height, weight, regular exercise and percent fat in a large-scale population-based study of Korean males and females aged ≥ 50 years.

## Methods

### Subjects

The Dong-gu Study is an ongoing prospective study designed to investigate the prevalence, incidence, and risk factors for chronic disease in an urban population[29]. To identify potential participants, the national resident registration records were used. From 2007 to 2010, 34,040 eligible subjects aged ≥50 years, and who resided in the Dong-gu district of Gwangju Metropolitan City in South Korea, were invited to participate by mail and telephone. A total of 9,260 subjects were enrolled (response rate: 27.2%; 3,711 male and 5,549 female). Of those, 9,206 subjects underwent lumbar spine or hip BMD using a Lunar Prodigy bone densitometer (GE, Madison, WI). 9,056 subjects had both lumbar spine and femoral neck BMD. Of those, 75 subjects were excluded because of missing data on waist circumference, anthropometric data and body composition. The final sample consisted of 8,981 individuals (3,592 men and 5,389 men). All participants provided informed consent, and the study was conducted in accordance with the guidelines in The Declaration of Helsinki. The study was approved by the institutional review board of Chonnam National University Hospital.

### Anthropometric measurements

Body weight, lean body mass, fat mass and fat percentage were measured in indoor clothing or light gown without shoes by bioelectrical impedance analysis using a calibrated Inbody 520 (Biospace Co. Korea). Height was measured to the nearest 0.1 cm, and weight was measured in the upright position to the nearest 0.1 kg. Waist circumference was measured with the subject standing, at the level midway between the lower rib margin and the iliac crest. Hip circumference was measured at the fullest point around the buttocks. Waist circumference was divided by hip circumference to calculate waist-to-hip ratio. Regular exercise was categorized as irregular or regular based on the frequency of recreational activity and exercise during a week.

### Measurements of bone mineral density

Participants had their lumbar spine and femoral neck BMD measured by Lunar Prodigy (GE, Madison, WI). The lumbar spine BMD represents the average BMD of L1-L4. Daily phantom scans were performed each morning for proper quality control. All BMD scans were conducted using standardized procedures following the manufacturer’s recommended protocols by well-trained examiners. Intrascanner reproducibility of repeated measurements, expressed as coefficient of variation, was less than 1%.

### Statistical analysis

The descriptive data for the major characteristics and the BMD values of the two groups are expressed as the mean ± standard deviation (SD). We used *t*-tests to determine statistical differences. Waist circumference was divided into sex-specific quartiles. Fractional polynomial regression was used to describe the association between weight, body fat percent, and waist circumference and BMD, and to evaluate the association between waist circumference and BMD after adjusting for age, height, weight, and body fat percent. Multiple linear regression analysis was used to evaluate the relationship between the quartiles of waist circumference and BMD, after adjusting for age, height, weight, and regular exercise in the first model, and further adjusting for body fat percent in the second model. In addition, multiple linear regression analyses were used to evaluate the linear relationship of waist circumference, waist/hip ratio, body fat percent and fat mass with BMD after adjusting for age, height, weight, and regular exercise. Statistical analyses were conducted using Stata version 12 (StataCorp, Texas, USA).

## Results

### Characteristics of the study subjects

**Figure 1:**
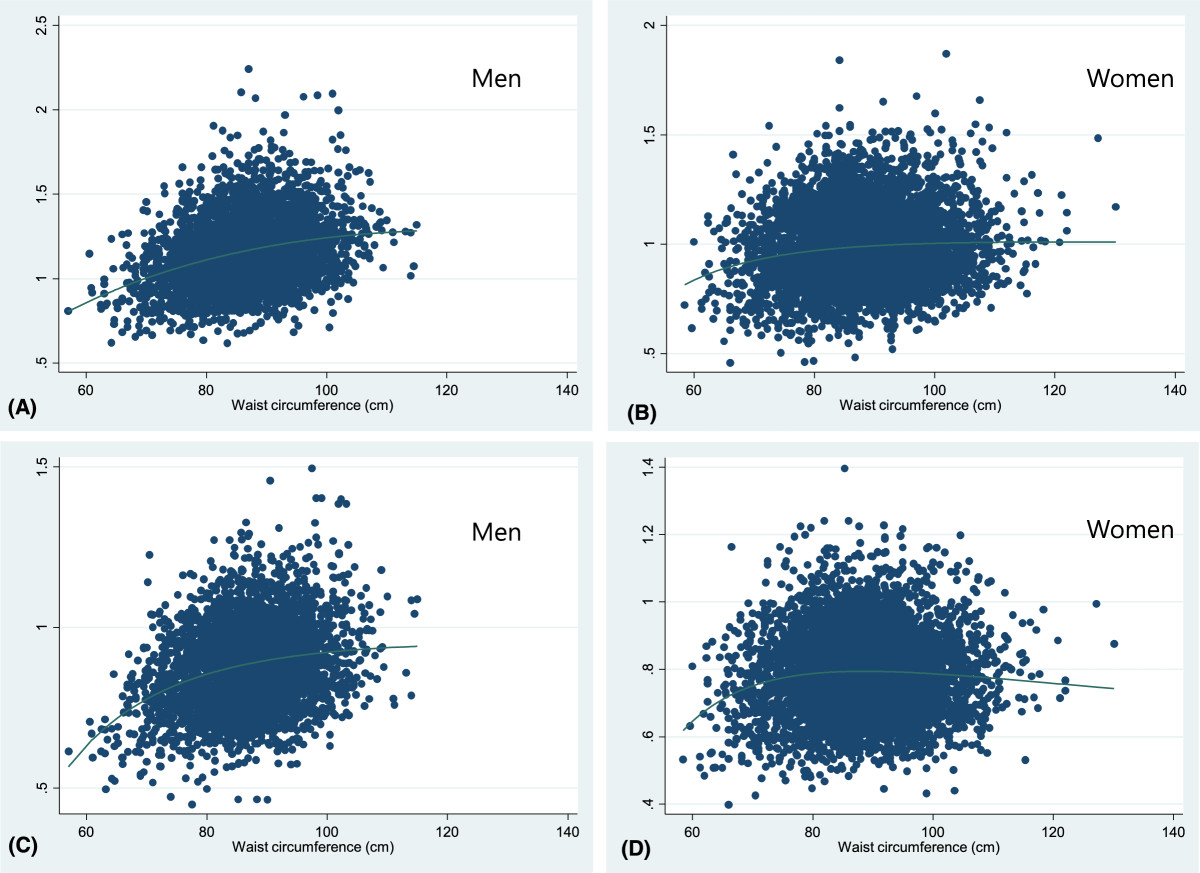
**Fractional polynomial regression line between waist circumference and BMD according to sex, before adjusting for covariates.** Lumbar spine **(A-B)**, Femoral neck **(C-D)**.

Table 1 shows the baseline characteristics of the subjects are shown in. The mean age at baseline was 66.2 ± 8.0 years for males and 64.4 ± 8.2 years for females. The mean height, weight, and BMI were respectively, 165.9 ± 5.7 cm, 65.9 ± 9.1 kg, and 23.9 ± 2.8 kg/m^2^ for males and 153.2 ± 5.5 cm, 57.8 ± 8.0 kg, and 24.6 ± 3.0 kg/m^2^ for females. The mean abdominal circumference was 87.1 ± 7.8 cm in males and 88.5 ± 9.0 cm in females. Waist to hip ratio, percent body fat, and fat mass were significantly higher in females than in males. The mean BMD at the lumbar spine and femoral neck sites was considerably higher in males than females.Figures 1 and2 show the fractional polynomial regression of waist circumference by BMD according to sex before and after adjustment for covariates. The bivariate analysis revealed a positive quadratic relationship between waist circumference and BMD with a plateau or slight decline at the higher levels. However, after adjusting for age, height, weight, and percent body fat, we observed a negative quadratic relationship between waist circumference and BMD with an initial incline; thus, the correlation between waist circumference and BMD values shifted from positive to negative after adjusting for the covariates.

**Table 1:** **Characteristics of the study subjects**

	Men (n = 3592)	Women (n = 5389)	p-value
Age (years)	66.2 ± 8.0	64.4 ± 8.2	<0.001
Height (cm)	165.9 ± 5.7	153.2 ± 5.5	<0.001
Weight (kg)	65.9 ± 9.1	57.8 ± 8.0	<0.001
BMI (kg/m^2^)	23.9 ± 2.8	24.6 ± 3.0	<0.001
Regular exercise (%)	1391 (38.7)	1521 (28.2)	<0.001
Abdominal circumference (cm)	87.1 ± 7.8	88.5 ± 9.0	<0.001
Waist to hip ratio	0.94 ± 0.05	0.95 ± 0.07	<0.001
Body fat percent (%)	25.8 ± 5.8	35.5 ± 5.9	<0.001
Fat mass (kg)	17.3 ± 5.5	20.8 ± 5.7	<0.001
Lumbar spine BMD (g/cm^2^)	1.161 ± 0.204	0.985 ± 0.169	<0.001
Femoral neck BMD (g/cm^2^)	0.881 ± 0.13	0.788 ± 0.122	<0.001

Values are mean ± SD or number (percentage).

BMI, body mass index; BMD, bone mineral density.

**Figure 2:**
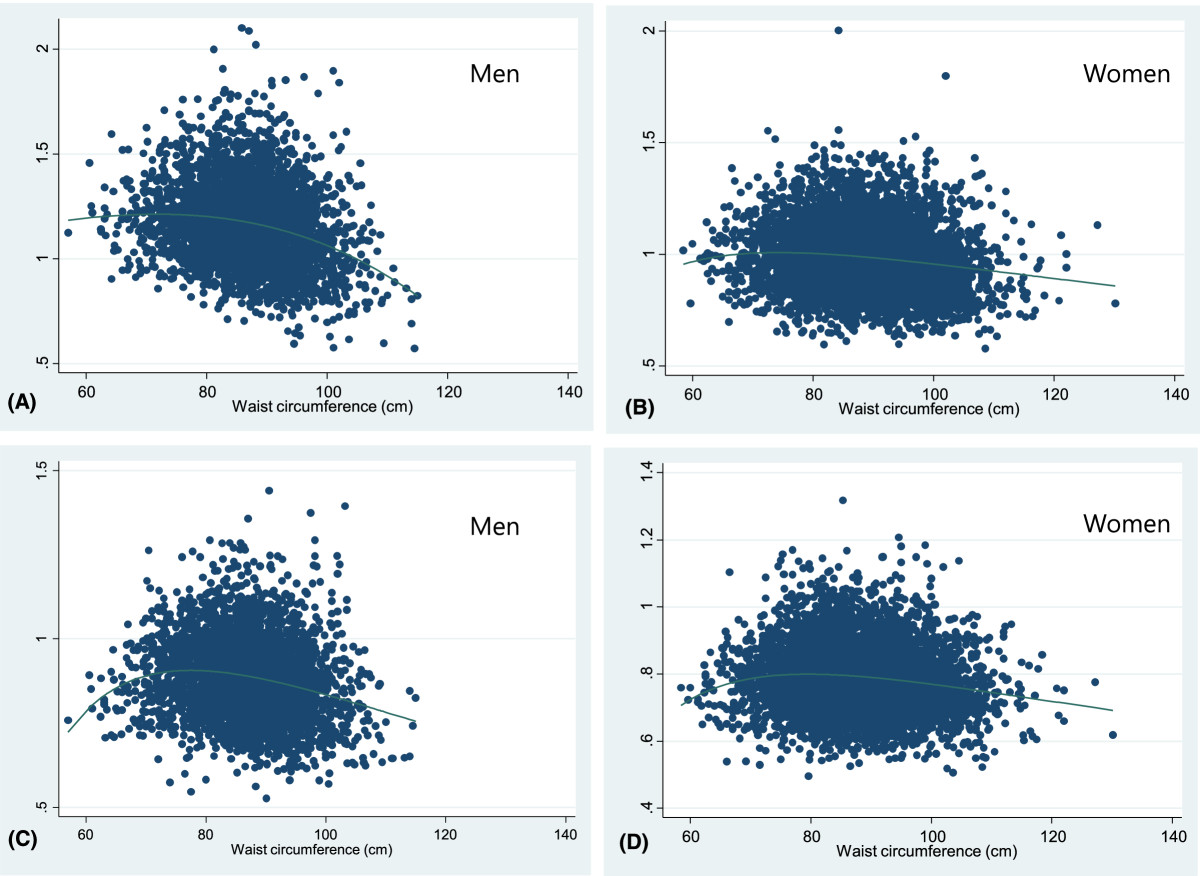
**Fractional polynomial regression line between waist circumference and BMD according to sex, adjusted for age, weight, height, body fat percent.** Lumbar spine **(A-B)**, Femoral neck **(C-D)**.

### Adjusted means of bone mineral density by quartiles of waist circumference

Table 2 shows femoral neck and lumbar spine BMD according to quartile of waist circumference. We found a negative linear association between waist circumference and BMD at both sites in males and females after adjusting for age, height, weight and regular exercise. Lumber spine BMD was lower in the third and fourth quartiles of waist circumference than in the first quartile in males and females. However, we found no significant difference between the first and second quartiles. Although further adjustment for percent body fat slightly attenuated these associations, they remained significant. We found interaction effects between sex and waist circumference for BMD at the lumbar spine and femoral neck sites (*P* = 0.001 and 0.016, respectively).

**Table 2:** **Adjusted means of bone mineral density by quartiles of waist circumference**

		Quartile of waist circumference					
		1st	2nd	3rd	4th	P for trend	P for interaction
Number (men/women)		898/1346	894/1355	896/1345	904/1343		
Range (men/women, cm)		(57.0-82.2)/(58.4-82.5)	(82.3-87.2)/(82.6-88.6)	(87.3-92.1)/(88.7-94.2)	(92.2-115.0)/(94.3-130.1)		
Model 1	Lumbar spine						
	Men	1.189 ± 0.009	1.185 ± 0.006	1.153 ± 0.006	1.117 ± 0.009	<0.001	
	Women	0.999 ± 0.005	0.996 ± 0.004	0.977 ± 0.004	0.967 ± 0.005	<0.001	0.001
	Femoral neck						
	Men	0.903 ± 0.005	0.896 ± 0.004	0.875 ± 0.004	0.851 ± 0.005	<0.001	
	Women	0.797 ± 0.003	0.794 ± 0.003	0.787 ± 0.003	0.772 ± 0.003	<0.001	0.008
Model 2	Lumbar spine						
	Men	1.185 ± 0.009	1.184 ± 0.006	1.154 ± 0.006	1.120 ± 0.009	<0.001	
	Women	0.998 ± 0.005	0.996 ± 0.004	0.978 ± 0.004	0.968 ± 0.005	<0.001	0.001
	Femoral neck						
	Men	0.896 ± 0.005	0.895 ± 0.004	0.877 ± 0.004	0.856 ± 0.005	<0.001	
	Women	0.794 ± 0.003	0.794 ± 0.003	0.788 ± 0.003	0.774 ± 0.003	<0.001	0.016

Values are mean difference with 1st quartile group in g/cm^2^ with 95% confidence interval, adjusted age, height, weight, and regular exercise (model 1) or age, height, weight, regular exercise and body fat percent (model 2).

### Linear association between obesity-related phenotypes and bone mineral density

Table 3 shows results of linear regression analysis of obesity-related phenotypes with BMD. Significant negative associations of waist circumference, waist/hip ratio, body fat percent and fat mass with BMD were found in both sexes.

**Table 3:** **Linear association between obesity-related phenotypes and bone mineral density**

			Unadjusted		Adjusted*		
			Coefficients	p-value	Coefficients	Standardized coefficients	p-value
Lumbar spine BMD	Men	Waist circumference (10 cm)	0.075 ± 0.004	<0.001	−0.051 ± 0.008	−0.198	<0.001
		Waist/hip ratio	0.575 ± 0.062	<0.001	−0.203 ± 0.070	−0.054	0.004
		Body fat percent (10%)	0.072 ± 0.006	<0.001	−0.024 ± 0.008	−0.067	0.003
		Fat mass (10 kg)	0.111 ± 0.006	<0.001	−0.049 ± 0.012	−0.132	<0.001
	Women	Waist circumference (10 cm)	0.021 ± 0.003	<0.001	−0.020 ± 0.004	−0.108	<0.001
		Waist/hip ratio	−0.113 ± 0.031	<0.001	−0.103 ± 0.031	−0.045	0.001
		Body fat percent (10%)	0.031 ± 0.004	<0.001	−0.016 ± 0.006	−0.054	0.007
		Fat mass (10 kg)	0.073 ± 0.004	<0.001	−0.041 ± 0.010	−0.138	<0.001
Femoral neck BMD	Men	Waist circumference (10 cm)	0.044 ± 0.003	<0.001	−0.028 ± 0.005	−0.170	0.035
		Waist/hip ratio	0.236 ± 0.040	<0.001	−0.136 ± 0.043	−0.056	0.002
		Body fat percent (10%)	0.023 ± 0.004	<0.001	−0.031 ± 0.005	−0.137	<0.001
		Fat mass (10 kg)	0.057 ± 0.004	<0.001	−0.053 ± 0.007	−0.223	<0.001
	Women	Waist circumference (10 cm)	0.005 ± 0.002	0.003	−0.012 ± 0.002	−0.085	0.030
		Waist/hip ratio	−0.209 ± 0.022	<0.001	−0.082 ± 0.020	−0.050	<0.001
		Body fat percent (10%)	0.003 ± 0.003	0.0349	−0.021 ± 0.004	−0.103	<0.001
		Fat mass (10 kg)	0.036 ± 0.003	<0.001	−0.052 ± 0.007	−0.242	<0.001

*Unstandardized regression coefficients and standardized regression coefficients were calculated by using a multiple linear regression adjusting for age, height, weight, and regular exercise separately for each obesity-related phenotype.

## Discussion

We investigated the association between waist circumference as an indicator of abdominal obesity and BMD in a large-scale population-based study of Korean males and females aged ≥50 years. Our results indicated that after adjusting for age, weight, height, regular exercise and percent body fat, waist circumference was negatively associated with lumbar spine and femoral neck BMD in older Koreans, particularly males.

The results of previous investigations of the association between surrogate or direct measures of central adiposity and BMD have been inconsistent. Three previous population-based studies that adjusted for the weight-bearing effect of body weight found that the waist-to-hip ratio (WHR) was negatively associated with BMD in the lumbar spine and calcaneus and with bone mineral content (BMC)[18–20], however, another study found a highly significant positive correlation between BMD in the proximal and ultradistal radius and WHR in obese individuals[30]. Moreover, several previous studies investigating the association between direct measures of central adiposity and BMD reported conflicting results. Two studies found that BMD was inversely associated with body-weight-adjusted abdominal fat mass and lean body mass-adjusted abdominal visceral adipose tissue (VAT)[31, 32], however, other studies reported positive relationship between abdominal fat distribution,percent truncal fat and bone mass and BMD[33, 34].

Some studies assessed the association between waist circumference as a metabolic syndrome components and BMD, but the results are also inconclusive. Three studies found a positive correlation between waist circumference and BMD[23–25], whereas others reported a negative correlation[22, 27, 28]. Moreover, general population-based studies have found a significant negative correlation between BMD and waist circumference in premenopausal females[35] and in males and females[36].

Several factors may account for these inconsistent results, such as differences in the populations under investigation (age, sex, and ethnicity), in the methods used to measure BMD and central adiposity, in sample size, or in the number and type of covariates controlled for across studies. The inclusion of body weight or BMI as a covariate may itself affect the association between central obesity and BMD. We assessed the relationship between waist circumference and BMD before and after adjusting for age, weight, height, percent body fat, and regular exercise, and found that the correlation changed from positive to negative after adjusting for these covariates. Several previous studies reported a positive correlation between fat mass and hip and spine BMD[21, 37] or total-body BMC[38] before adjusting for body weight; however, the association was negative after adjusting for body weight. Similarly, two studies in Korean males and postmenopausal females found a negative association between WHR and BMD in the calcaneus[19] or lumbar spine[18] after adjusting for BMI or body weight, whereas a study that did not adjust for body weight found a positive correlation between truncal fat mass and total hip and the femoral neck BMD in healthy premenopausal females[39]. Aghaei Meybodi et al.[40] did not adjust for weight and identified a positive relationship between all anthropometric measures and BMD in both sexes.

A greater body weight is thought to increase skeletal loading, which activates an adaptive response leading to an increase in bone density. Fat mass is a major component of body weight. When the mechanical loading effect of body weight is statistically removed, fat mass is negatively associated with bone. After controlling for age, body weight, height, and regular exercises, we identified a negative correlation between waist circumference and BMD in the femoral neck and lumbar spine in middle-aged and older males and females. Further adjustment for percent body fat slightly attenuated the correlations; however, they remained significant. Our findings suggest that weight-adjusted abdominal fat mass may have non-mechanical loading effects on bone mass and, thus, abdominal obesity may not always protect against osteoporosis. The negative effect of weight–adjusted abdominal fat mass on bone might be driven by higher levels of pro-inflammatory cytokines, which may up-regulate receptor activators of nuclear factor-kB ligand, leading to increased bone resorption and decreased BMD[41, 42].

Our results revealed a significant sex-related difference in waist circumference and BMD, such that the negative correlation between waist circumference and lumbar spine and femoral neck BMD was greater in males than in females. The reasons for this are not entirely clear, although hormonal differences may be an important factor underlying this effect. However, sex-related differences in the relationship between body fat and BMD are controversial. Katzmarzyk et al.[32] found no sex-related difference between BMD and VAT and abdominal subcutaneous (SAT) adipose tissue in African–American and white males and females, whereas another study found a positive relationship between fat mass femoral neck BMD in white and black females, but no significant relationship in males[43]. In contrast, Kim et al.[28] found a negative association between waist circumference and femoral neck BMD in males and females, particularly in males, which is consistent with our findings.

Our study had several strengths. To our knowledge, it is the largest investigation of the association between waist circumference and BMD in community-dwelling individuals (*n* = 8982). Furthermore, we controlled for multiple covariates and the study population included both males and females. Our study also had several limitations. First, we did not examine various inflammatory markers and diet information. Second, we did not examine the abdominal adiposity distribution, we could not determine the individual associations of VAT and SAT with BMD. Third, the cross-sectional design of our study did not allow us to establish causal relationships. Further investigation should examine the biological link between inflammation and waist circumference in the progression of osteoporosis.

## Conclusions

Waist circumference is independently and inversely associated with BMD when the body components are controlled for, suggesting that waist circumference is a potential predictor of osteoporosis in middle-aged and older Korean males and postmenopausal females.

## Authors’ original submitted files for images

Below are the links to the authors’ original submitted files for images. Authors’ original file for figure 1 Authors’ original file for figure 2
