# Supplementary figures and images for: Sex-related differences in the association between waist circumference and bone mineral density in a Korean population
Source: BMC Musculoskelet Disord. 2014 Oct 2;15:326. doi: 10.1186/1471-2474-15-326 (PMC4193133; doi:10.1186/1471-2474-15-326)

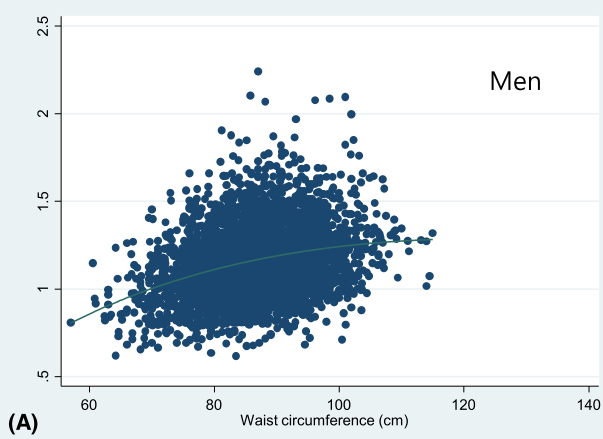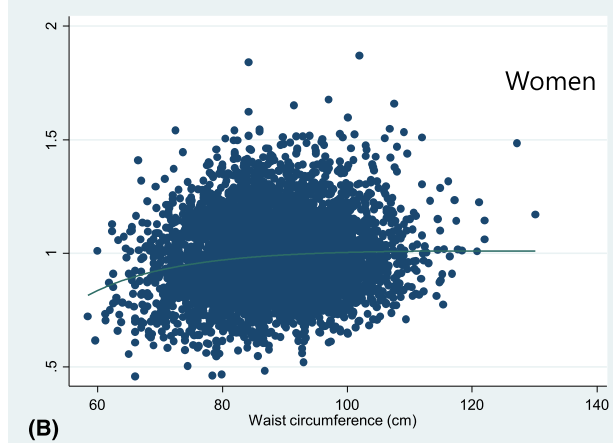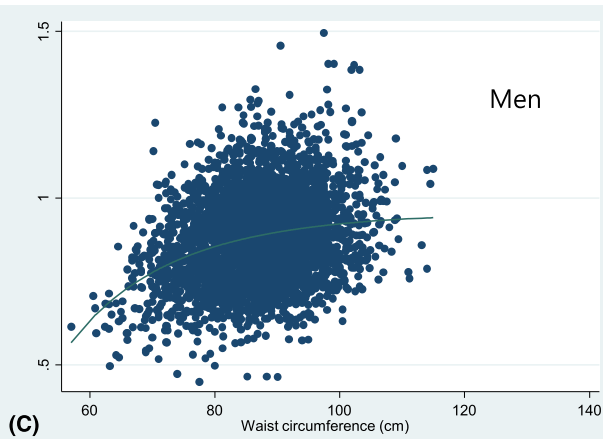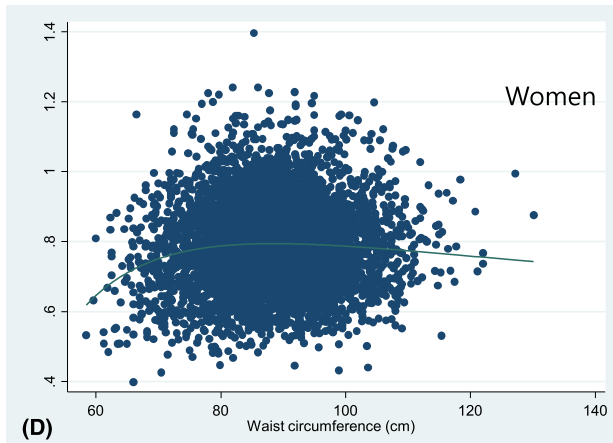

Supplement: Supplementary file 1 — Authors’ original file for figure 1 [file 12891_2014_2265_MOESM1_ESM.pdf]

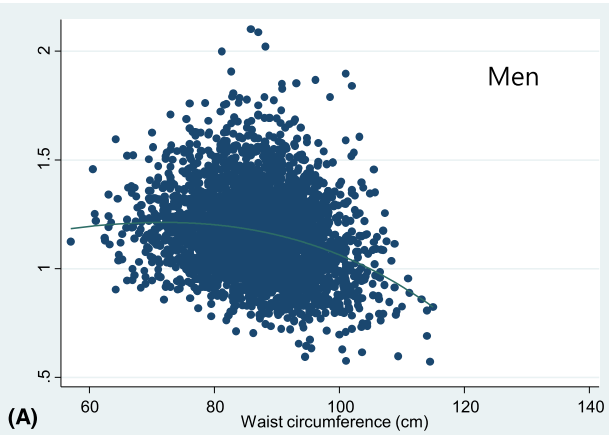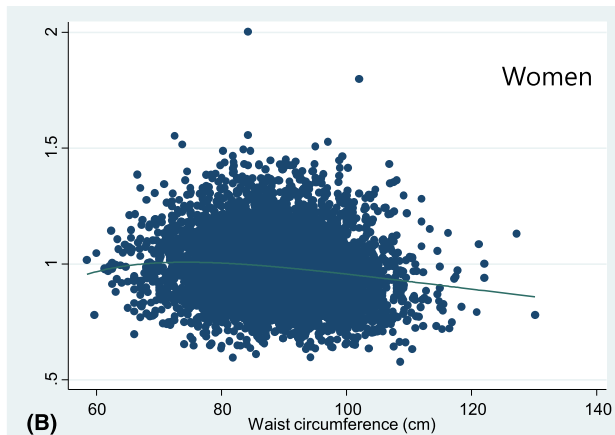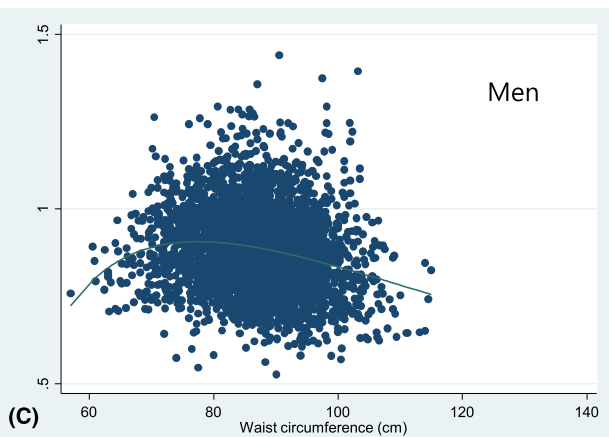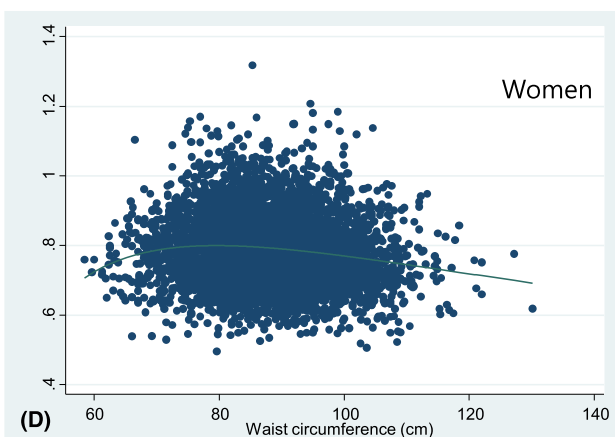

Supplement: Supplementary file 2 — Authors’ original file for figure 2 [file 12891_2014_2265_MOESM2_ESM.pdf]
